# Over Time Changes in the Transcriptomic Profiles of Tomato Plants with or Without *Mi-1* Gene During Their Incompatible or Compatible Interactions with the Whitefly *Bemisia tabaci*

**DOI:** 10.3390/plants14071054

**Published:** 2025-03-28

**Authors:** Susana Pascual, Clara I. Rodríguez-Álvarez, Irene López-Vidriero, José M. Franco-Zorrilla, Gloria Nombela

**Affiliations:** 1 Entomology Group, Plant Protection Department, Instituto Nacional de Investigación y Tecnología Agraria y Alimentaria (INIA), Spanish National Research Council (CSIC), Ctra. Coruña km 7, 28040 Madrid, Spain; 2 Department of Plant Protection, Institute for Agricultural Sciences (ICA), Spanish National Research Council (CSIC), Serrano 115 Dpdo., 28006 Madrid, Spain; clara.rodriguez@csic.es (C.I.R.-Á.); gnombela@ica.csic.es (G.N.); 3 Genomics Unit, Centro Nacional de Biotecnología (CNB), Spanish National Research Council (CSIC), Darwin 3, 28049 Madrid, Spain; irene.lopez@aei.gob.es (I.L.-V.); jmfranco@cnb.csic.es (J.M.F.-Z.)

**Keywords:** *Bemisia tabaci*, defence-related genes, Mapman, *Mi-1* mediated resistance, plant defence, plant resistance, sol genomics network, tomato, transcriptional profile, whiteflies

## Abstract

Understanding the resistance mechanisms of plants against pests contributes to the sustainable deployment of plant resistance in Integrated Pest Management (IPM) programmes. The *Mi-1* gene in tomato is the only one described with the capacity to provide resistance to different types of harmful organisms such as plant parasitic nematodes and pest insects, including the whitefly *Bemisia tabaci* MED (Mediterranean species). In this work, gene expression in the interaction of *B. tabaci* with susceptible tomato plants lacking the *Mi-1* gene (cv. Moneymaker, compatible interaction), and with resistant plants carrying the *Mi-1* gene (cv. Motelle, incompatible interaction) was studied using the oligonucleotide microarray technique. Both interactions were studied 2 and 12 days post infestation (dpi) of plants with adult insects. At 2 dpi, 159 overexpressed and 189 repressed transcripts were detected in the incompatible interaction, while these figures were 32 and 47 in the compatible one. Transcriptional reprogramming was more intense at 12 dpi but, as at 2 dpi, the number of transcripts overexpressed and repressed was higher in the incompatible (595 and 437, respectively) than in the compatible (71 and 52, respectively) interaction. According to the Mapman classification, these transcripts corresponded mainly to genes in the protein and RNA categories, some of which are involved in the defence response (signalling, respiratory burst, regulation of transcription, PRs, HSPs, cell wall or hormone signalling). These results provide a wealth of information about possible genes related to the resistance provided by the *Mi-1* gene to *B. tabaci*, and whose role deserves further investigation.

## 1. Introduction

Pest control in the current context of intensified agriculture continues to be dominated by chemical methods, despite its disadvantages for human and environmental health. The adoption of integrated pest management (IPM) is urgently needed to contribute to sustainable, resilient, profitable and robust agricultural systems, and plant resistance to insect pests is a fundamental element in IPM implementation. The *Mi-1* gene of tomato is a unique resistance gene conferring resistance to diverse organisms, such as root-knot nematodes (*Meloidogyne* spp.) [1], the potato aphid *Macrosiphum euphorbiae* [2], the tomato psyllid *Bactericera cockerelli* [3] and the insect studied in the present work, the whitefly *Bemisia tabaci* (Hemiptera; Aleyrodidae) [4]. *B. tabaci* is amongst the most damaging agricultural pest species worldwide, because of its wide host range, resistance to insecticides and virus transmission capacity [5,6,7].

*Mi-1* mediated resistance works by following a gene-by-gene interaction, resulting in a hypersensitive reaction (HR) in the case of nematodes [8], but not in the case of aphids [9] or whiteflies [10]. This gene was introduced to cultivated tomato from the wild relative *Lycopersicum peruvianum* [11], and like other resistance genes, is a Coiled coil domain-nucleotide binding site-leucine rich repeat (CC-NBS-LRR) gene [12]. For *Mi-1* functioning against nematodes, aphids, and whiteflies, the gene *Rme1* is required [13,14]. In the case of aphids, Mitogen Activated Protein Kinases (MAPK) are also required [15], as well as the transmembrane kinase (SERK1) [16]. *Hsp90* and *Sgt1* genes are also required for *Mi-1* mediated resistance to aphids and nematodes [17] and *Hsp90* is required also for *Mi-1*-mediated resistance of tomato to *B. tabaci* [18].

Previous research by our group has investigated the basal differences in the transcriptomes of plants with and without the *Mi-1* gene, and the changes in these differences after *B. tabaci* infestation [10]. Ten transcripts were expressed at least twofold in uninfested *Mi-1*-carrying plants (cv. Motelle) than in plants lacking this gene (cv. Moneymaker), while other eight transcripts were expressed half or less. After whitefly infestation, differences between Motelle and Moneymaker cultivars increased up to 14 transcripts more expressed in Motelle than in Moneymaker and 14 transcripts less expressed. However, to better understand the potential role of different genes in the *Mi-1* mediated resistance to whiteflies, it is necessary to study the expression differences between infested and non-infested plants of the same tomato genotype, separately during each of the compatible (Moneymaker) and incompatible (Motelle) interactions of tomato with *B. tabaci*.

A great number of works have studied compatible interactions of susceptible plants with pathogens [19,20] and insects [21,22]. Transcriptomic analyses have been performed on tomato during the compatible and incompatible interaction with pathogens, such as *Xanthomonas campestris* pv. *vesicatoria* [23,24], nematodes [25,26] or Tomato Yellow Leaf Curl Virus (TYLCV) [27]. In the case of insect pests, the transcriptomic profiles of partially resistant and susceptible tomato against *Tuta absoluta* have been compared [28]. As for whiteflies and tomato in particular, only the compatible interaction was previously studied [29]. Other works focused on the compatible interactions of whiteflies with other plant species such as *Arabidopsis* [30] and *Physalis philadelphica* [31]. Whiteflies are included in the review by Thompson and Goggin (2006) on compatible and incompatible interactions of different plants with phloem-feeding insects [32]. In other systems, however, gene expression has been studied in incompatible interactions, such is the case of *Glycine max-Aphis glycines* [33], *Oryza sativa*-*Orseolia oryzae* [34], *Solanum tuberosum*-*Phytophthora infestans* [35], *Brassica juncea-Aphis craccivora* [36], *Beta vulgaris* subsp. *vulgaris*-*Heterodera schachtii* [37] *or Arabidopsis-Alternaria brassicicola* [38].

The objective of this work is to deepen the knowledge of both, compatible (susceptibility) and incompatible (*Mi-1*-mediated resistance) interactions of tomato with the whitefly *B. tabaci* through differential gene expression analysis using the GeneChip™ Tomato Genome Array (Affymetrix^®^, Santa Clara, CA, USA). To study the incompatible interaction, differences were searched between the transcriptional profiles of infested and uninfested adult plants of the *Mi-1*-bearing cultivar Motelle. Similarly, the transcriptomes of infested and uninfested plants of the susceptible cultivar Moneymaker (lacking the *Mi-1* gene) were compared to analyze the compatible interaction. Two different time points were considered (2 and 12 days after infestation) to understand the transcriptional changes produced over time, at early and late stages of each interaction.

## 2. Results

A complete file with the lists of tomato transcripts differentially expressed during the tomato/*B. tabaci* interactions is included as Supplementary Materials Table S1. Tomato transcripts differentially expressed in the compatible interaction with *B. tabaci* were obtained when comparing infested and uninfested plants of the susceptible cultivar Moneymaker. Similarly, differential transcripts detected during the incompatible interaction were obtained by comparing infested and uninfested plants of cv. Motelle (carrying the *Mi-1* gene). For each interaction type, the results of the analyses carried out two time points are shown: first at 2 days post-infestation (2 dpi) and again later at 12 dpi.

### 2.1. The Compatible Interaction Tomato/Whitefly: Transcriptional Reprogramming in Moneymaker in Two Phases of the Infestation by B. tabaci

**Figure 1:**
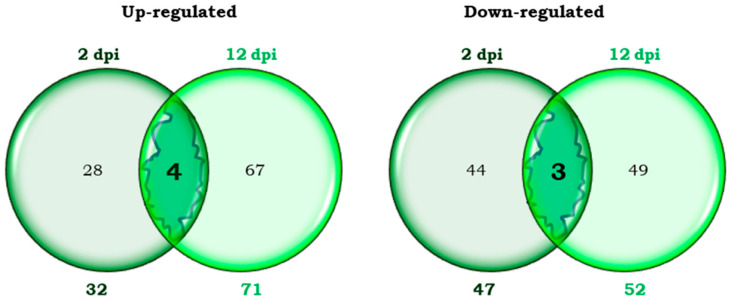
Venn diagram showing the number of transcripts differentially expressed in the tomato-*Bemisia tabaci* compatible (Moneymaker) interaction at 2 and 12 dpi. The overlap are transcripts equally found at both time points. Only transcripts with statistically significant relative expression values (false discovery rate (FDR) < 0.05)) and fold change (FC) ≥ 2 (up) or ≤−2 (down) are included.

In the early phase of infestation (2 dpi), 32 transcripts were more expressed (up-regulated), and 47 transcripts were less expressed (down-regulated) in Moneymaker infested plants than in uninfested plants of the same cultivar. In a later phase (12 dpi), 71 up-regulated and 52 down-regulated transcripts were detected in the infested plants compared to the uninfested plants. These numbers are represented by Venn diagrams (Figure 1). Only 7 transcripts were differentially expressed at both 2 and 12 dpi. On the contrary, the great majority were exclusive to one or the other of these two phases of the infestation.

The range of expression of the overexpressed transcripts was similar at 12 dpi (2- to 6-fold higher in infested than in uninfested plants) than at 2 dpi (2- to 4-fold higher in infested versus uninfested plants). While for repressed transcripts, the range of expression was slightly higher at 2 dpi (2- to 9-fold lower in infested than in non-infested plants) than at 12 dpi (only 2- to 4-fold lower in infested than in non-infested plants).

**Figure 2:**
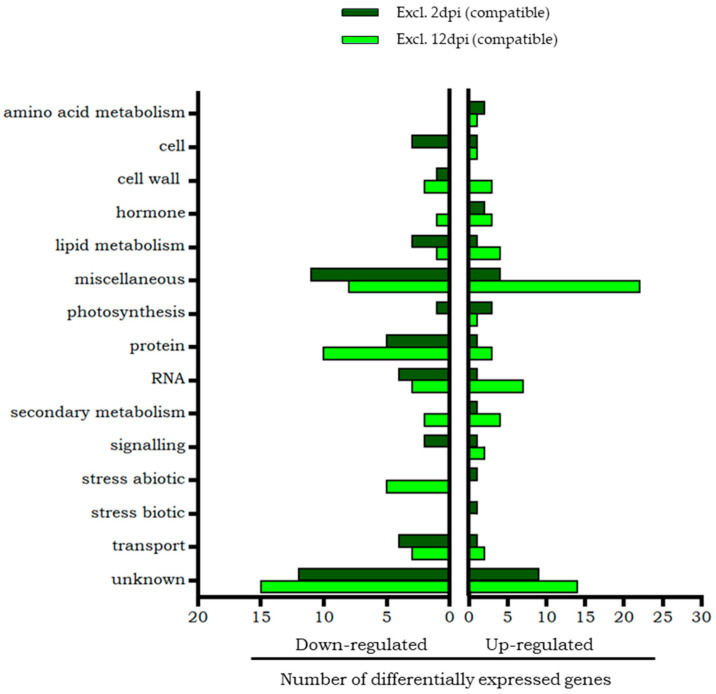
Functional classification by Mapman of the differential transcripts in the compatible interaction, exclusive at 2 dpi (dark green) and exclusive at 12 dpi (light green) according to the biological processes in which they intervene.

The classification of the differential transcripts exclusive of the compatible interaction at 2 dpi and 12 dpi according to Mapman is shown in Figure 2. Genes common to both phases are not included in this figure because they were scarce and are individually described below. The most numerous categories are those that include genes with unknown function or whose function is known but Mapman does not separate them into individual categories (miscellaneous group).

#### 2.1.1. Differential Transcripts of Moneymaker Tomato Common to Both Early and Late Phases of *B. tabaci* Infestation

Only 4 transcripts were up-regulated at both 2 and 12 dpi (Figure 1), representing approximately 12% of the overexpressed genes at 2 dpi and 5.6% of those up-regulated at 12 dpi. The percentage representing the 3 down-regulated genes was just over 6% of the repressed transcripts at 2 dpi and 6% at 12 dpi.

Two of the 4 overexpressed transcripts corresponded to 2 genes encoding proteins related to defence processes (Glucan endo-1,3-beta-glucosidase and glutathione S-transferase). The 2 other transcripts represented genes not involved in stress response. No information was obtained from one of the 3 repressed transcripts, and the two other genes correspond to senescence regulators. The genes are described in the Supplementary Materials Table S1.

#### 2.1.2. Differential Transcripts of Moneymaker Tomato Unique to the Early Phase of Infestation (2 dpi)

The number of differentially expressed transcripts exclusively at 2 dpi in the compatible interaction were 72: 28 and 44 up-regulated and down-regulated transcripts, respectively (Figure 1). The expression level of the 28 overexpressed transcripts ranged from 2 to 4 times higher in the infested Moneymaker plants than in the uninfested ones. The expression level of the 44 repressed transcripts was 2 to 9-fold lower in the infested plants than in the uninfested plants.

According to the Mapman functional classification (Figure 2), a higher representation of overexpressed genes than repressed genes was observed in the categories of amino acid metabolism, hormone metabolism (which included genes involved in ethylene metabolism, one of them of response to molecule of fungal origin), secondary metabolism, and photosynthesis. Among the overexpressed genes, the chaperone Hsp70, included in the abiotic stress category, and the pathogenesis related PR-1 protein, in the biotic stress category, stand out. Although in the miscellaneous group, a glucan endo-1,3-beta-glucosidase, involved in defence response, was overexpressed.

On the other hand, more repressed than overexpressed genes were found in the categories of cell processes, cell wall, lipid metabolism (in which phospholipase PLDβ2), related to stress response, stands out), protein (with a proteinase inhibitor), RNA (represented by several families of transcription factors), signalling and transport. It is worth mentioning the repression of the enzyme Nicotinamide adenine dinucleotide phosphate (NADPH) protochlorophyll oxidoreductase, involved in the last steps of chlorophyll biosynthesis, whose role is vital in photosynthesis.

#### 2.1.3. Differential Transcripts of Moneymaker Tomato Unique to the Late Phase of Infestation (12 dpi)

In the compatible interaction, 67 and 49 transcripts were up-regulated and down-regulated, respectively, only at 12 dpi (Figure 1). The expression level of the 67 overexpressed transcripts was between 2 and 6 times higher in infested than in uninfested plants. The 49 exclusively repressed transcripts at 12 dpi were expressed 2–4 fold less in infested than in uninfested plants.

In general, a greater representation of overexpressed than repressed genes were obtained in most categories (Figure 2), being remarkable the RNA category (which included the transcription factors WRKY and MYB (myeloblastosis), and the hormone metabolism category (specifically, ethylene metabolism). It is also worth mentioning the cell wall category (which included several xyloglucan endotransglucosylase-hydrolase (XTH) enzymes) and finally, the secondary metabolism category that grouped overexpressed genes related to flavonoid metabolism, including the gene that encodes the enzyme chalcone synthase.

The categories with the highest number of down-regulated transcripts were transport, protein, and abiotic stress. In fact, the latter grouped only repressed genes, some of which encoded different classes of small chaperones. Finally, it is highlighted the repression of a gene involved in defence processes that encodes the enzyme that inhibits pathogenic endoglucanases, which was not assigned by Mapman to any known category.

### 2.2. The Incompatible Interaction Tomato/Whitefly: Transcriptional Reprogramming in Motelle in Two Phases of the Infestation by B. tabaci

**Figure 3:**
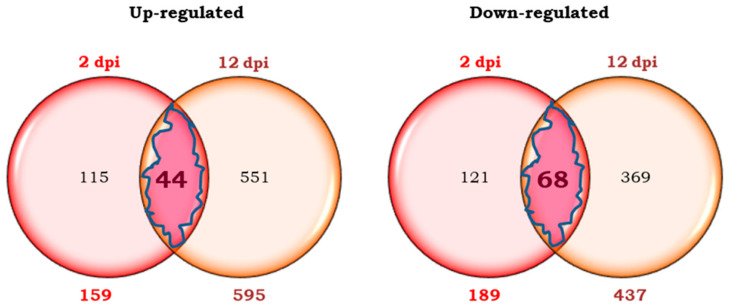
Venn diagram showing the number of transcripts differentially expressed in the tomato-*Bemisia tabaci* incompatible (Motelle) interaction at 2 and 12 dpi. The overlap are transcripts equally found both time points. Only transcripts with statistically significant relative expression values (FDR < 0.05) and fold change (FC) ≥ 2 (up) or ≤−2 (down) are included.

In the early phase of infestation (2 dpi), 159 transcripts were more expressed (up-regulated), and 189 transcripts were less expressed (down-regulated) in Motelle infested plants than in uninfested plants of the same cultivar. In a later phase (12 dpi), 595 up-regulated and 437 down-regulated transcripts were detected in the infested plants compared to the uninfested plants. These numbers are represented by Venn diagrams (Figure 3).

The range of differential expression of these transcripts was much greater at 12 than at 2 dpi: at 2 dpi, the up-regulated transcripts were 2- to 24-fold more expressed in the infested than in the uninfested Motelle plants, and the down-regulated transcripts were 2- to 14-fold less expressed in infested versus uninfested plants. In contrast, at 12 dpi, the up-regulated transcripts were 2 to 51-fold more expressed in the infested than in the uninfested plants, and the down-regulated transcripts were 2- to 87-fold less expressed in the infested than in the uninfested plants.

**Figure 4:**
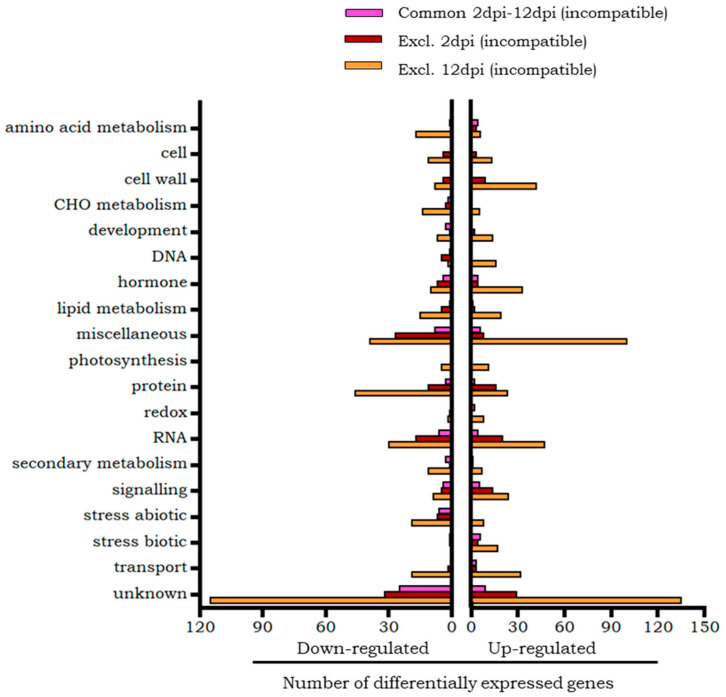
Functional classification by Mapman of the differential transcripts in the incompatible interaction, exclusive at 2 dpi (dark red), exclusive at 12 dpi (orange) and those in common (pink), according to the biological processes in which they intervene.

The transcripts of the incompatible interaction differentially expressed only at 2 dpi or at 12 dpi, as well as those common to both time points, were grouped according to the biological processes in which they intervene (Figure 4). The categories with most transcripts are those that include genes with unknown function or that are in the miscellaneous group.

#### 2.2.1. Differential Transcripts of Motelle Tomato Common to Both Early and Late Phases of *B. tabaci* Infestation

In the incompatible interaction, 44 up-regulated and 68 down-regulated transcripts, common at 2 and 12 dpi, were obtained. They represented approximately 28% of the transcripts overexpressed at 2 dpi and 7% of the number of transcripts up-regulated at 12 dpi. The percentages of the down-regulated transcripts represented 36% and 16% of the transcripts repressed at 2 dpi and 12 dpi, respectively.

According to the Mapman classification (Figure 4), a greater representation of overexpressed genes was observed in the signalling and biotic stress categories. In the signalling category, calmodulin, calmodulin-binding protein herbivore elicitor-regulated and MAPK3 stand out. In the biotic stress category, several PRs were overexpressed, including the *Ve* gene. In the RNA category, it is worth highlighting the overexpression of transcription factors of the MYB and WRKY families, although this category includes a greater number of repressed than overexpressed transcripts.

Only one repressed gene, (NADPH oxidase) was assigned to the biotic stress category. All genes in the abiotic stress category were repressed, including genes encoding different classes of small chaperones. In the hormone category, the same number of overexpressed and repressed genes was found, including a PR transcriptional activator which was overexpressed. Several β1,3-glucan hydrolases, related to defence, were overexpressed, although Mapman assigned then to the miscellaneous category. Also, a repressed gene related to response to abscisic acid and water deprivation, was classified in the unknown category. The rest of the categories had a greater number of repressed than overexpressed genes. The gene encoding the ELI3 (Cinnamyl alcohol dehydrogenase) protein, in the category of secondary metabolism, was repressed.

#### 2.2.2. Differential Transcripts of Motelle Tomato Unique to the Early Phase of Infestation (2 dpi)

The number of transcripts differentially expressed in the incompatible interaction at 2 dpi (but not later) were 115 up-regulated and 121 down-regulated transcripts, (Figure 3). The expression level of the 115-overexpressed transcripts was between 2 and 24 times higher in infested than in uninfested Motelle plants. The expression level of the 121 was 2 to 14-fold lower in infested than in uninfested plants.

The Mapman categories (Figure 4) with the greatest representation were those of RNA and protein, with the overexpression of the WRKY and Ethylene Responsive (ERF) transcription factors standing out in the RNA category. In the signalling category, more overexpressed than repressed genes were found, highlighting the presence of calmodulins and the phi-1 (exordium-like) protein, whose expression is induced in response to stress. Also, in the cell wall category more overexpressed than repressed genes were found, including the enzymes XTH. In the biotic stress category, a greater number of overexpressed genes were also found, some of them coding for PRs different from the proteins common to both phases of the infestation.

Only down-regulated genes were found in the abiotic stress category, among which were genes encoding chaperones. Included in the protein category was a down-regulated gene encoding copine protein, a Ca^2+^-dependent phospholipid-binding protein. In the category of hormonal metabolism, a greater number of repressed genes were grouped, which were related to processes of synthesis or degradation of different hormones, compared to a smaller number of overexpressed genes, which were related to the transduction of the ethylene signal and to plant defence. The lipid metabolism category was made up of a greater number of repressed genes, among which phospholipase PLDb2, involved in stress responses, stands out. In the miscellaneous category, several Glucan endo-1,3-beta-glucosidases were repressed.

#### 2.2.3. Differential Transcripts of Motelle Tomato Unique to the Late Phase of Infestation (12 dpi)

The numbers of up-regulated and down-regulated transcripts only at 12 dpi of the incompatible interaction were 551 and 369, respectively (Figure 3). The expression level of the 551 up-regulated transcripts was between 2 and 51 times higher in infested than in uninfested plants. The 369 down-regulated transcripts were expressed between 2 and 87 times less in the infested than in the non-infested plants.

One of the categories with the greatest representation of differential genes was the RNA category, which included similar numbers of overexpressed and repressed genes (Figure 4). In the group of overexpressed genes, those that encoded for different families of transcription factors stood out, among which were MYB and WRKYs, such as CONSTANS interacting proteins, and bHLH transcription factors, related to abiotic stress. Among the repressed genes of this same RNA category, some that code for other transcription factors were included, highlighting those of the MADS-box family.

Most of the remaining overexpressed genes were grouped in the cell wall and hormone metabolism categories. Remarkable are various XTH enzymes, expansins, extension-likes and pectinesterases in the cell wall category, and numerous genes involved in different signalling pathways, such as the ethylene and gibberellins pathways in the hormone metabolism category. In the gibberellins pathways, the overexpression of the DELLA protein Gibberellic-Acid Insensitive (GAI) stands out. The signalling category also harboured a greater number of overexpressed than repressed genes, with Ca^2+^ signalling proteins other than calmodulin being found. The biotic stress category had only overexpressed genes, among which genes encoding PR and proteinase inhibitors were found. Finally, among the up-regulated transcripts, the gene encoding acid phosphatase 1 stands out, linked to the *Mi-1* gene, but included in the miscellaneous category according to Mapman classification.

The categories that grouped the highest number of down-regulated transcripts were protein and the abiotic stress category, which included genes encoding small chaperones. Among the repressed genes, a gene involved in defence processes that encodes the enzyme that inhibits pathogenic endoglucanases (xyloglucan endoglucanase inhibitor) stands out, although assigned to the unknown category.

To obtain further insight about the functional significance of the compatible and incompatible interactions, we performed a Gene Ontology (GO) term enrichment analysis of the exclusive differentially expressed gene lists (Supplementary Materials Figure S1). Genes down-regulated in the incompatible interaction were enriched in several GO terms (Biological Process, BP) related to primary and secondary metabolism, whereas down-regulation in the compatible interaction was associated with oxidation-reduction processes. In the case of up-regulated genes, only those derived from the incompatible interaction showed significant enrichment in Molecular Function GO terms, related to hydrolase and oxidoreductase activities (Supplementary Materials Figure S1).

### 2.3. Similarities and Differences Between Compatible and Incompatible Interactions in the Early Phase of B. tabaci Infestation (2 dpi)

After individually studying the compatible and the incompatible interactions of tomato with *B. tabaci*, the results obtained allowed a comparison of the transcriptional reprogramming in Moneymaker and Motelle, at each of the early and late phases of infestation separately.

**Figure 5:**
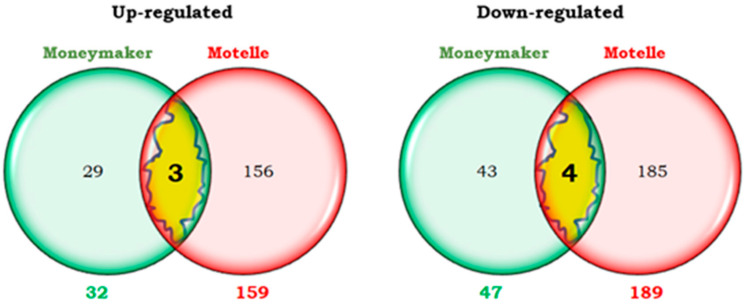
Venn diagram showing the number of transcripts differentially expressed in the tomato-*Bemisia tabaci* compatible (Moneymaker) and incompatible (Motelle) interactions at 2 dpi. The overlap are transcripts equally found in both interactions. Up-regulated represent transcripts more expressed in infested than in non-infested (control) plants. Down-regulated represent transcripts less expressed in infested than in non-infested (control) plants. Only transcripts with statistically significant relative expression values (FDR < 0.05) and fold change (FC) ≥ 2 (up) or ≤−2 (down) are included.

At the early phase of infestation (2 dpi) the numbers of transcripts differentially expressed between infested and uninfested plants of each tomato cultivar are shown by means of Venn diagrams (Figure 5). A greater number of transcripts differentially expressed was detected in the incompatible interaction (348 = 159 up + 189 down) than in the compatible interaction (79 = 32 up + 47 down). In both cases, the number of down-regulated transcripts was higher than that of up-regulated ones.

It is noteworthy that most of these differentially expressed transcripts at 2 dpi were unique to one or the other interaction, with only 7 transcripts (representing less than 2% of the total up- or down-regulated transcripts) common to compatible and incompatible interactions.

**Figure 6:**
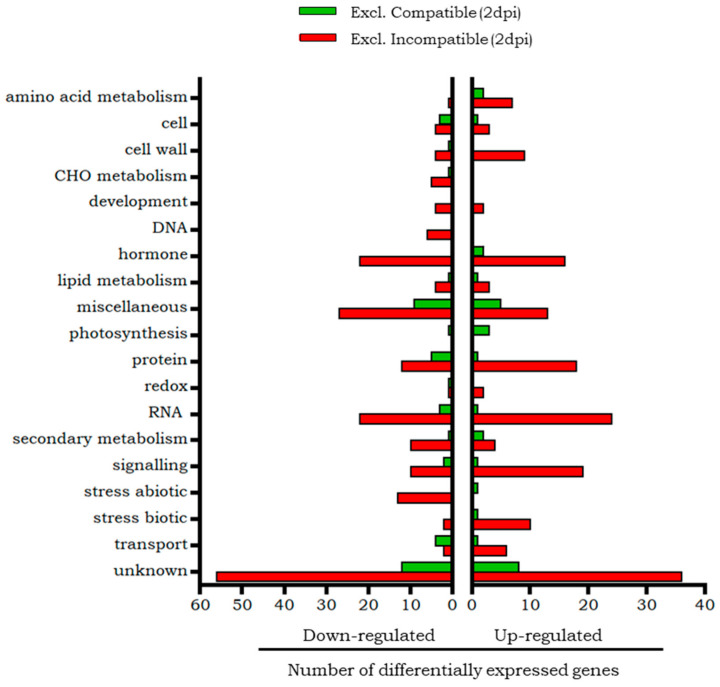
Functional classification by Mapman of the differential transcripts at 2 dpi, exclusive of the compatible interaction (green) and exclusive of the incompatible interaction (red), according to the biological processes in which they intervene.

Figure 6 shows the Mapman classification of the differential transcripts exclusive of the compatible interaction and those exclusive of the incompatible interaction, grouped according to the biological processes in which they intervene. The “unknown” group was the most represented in both interactions and was composed of transcripts whose biological processes are unknown.

#### 2.3.1. Transcripts Differentially Expressed at 2 dpi in Both Compatible and Incompatible Interactions

Only 3 up-regulated transcripts common to both interactions were obtained (Figure 5), accounting for approximately 2% of the up-regulated transcripts in the incompatible interaction and somewhat less than 9.5% in the compatible. One of this 3 transcripts encoded a glucan endo-1,3-β-glucosidase implicated in the defence of plants against pathogens. The other two transcripts corresponded to the PGPS/D12 gene related to oxidative response (protein plant cadmium resistance 2-like), and to a transmembrane protein.

The 4 common down-regulated transcripts represented 2% of the down-regulated transcripts in the incompatible interaction and 8.5% in the compatible. Among them, a transcription factor (Nuclear transcription factor Y subunit B-3) and one transcript related to the hypersensitive response (Hypersensitive response assisting protein) were identified. Fold changes for these transcripts differentially expressed at 2 dpi were similar for compatible and incompatible interactions and ranged between −3.98 and 2.61.

#### 2.3.2. Transcripts Differentially Expressed at 2 dpi Only in the Compatible Interaction

A total of 72 transcripts differentially expressed were detected in the compatible interaction but not in the incompatible one (Figure 5). Of them, the expression level of the 29 up-regulated transcripts ranged from 2 to 4-fold (Fold Change or FC) higher in Moneymaker-infested plants than in non-infested plants of the same cultivar.

Regarding the 43 down-regulated transcripts, their expression level was 2 to 9 times lower in infested plants than in plants without infestation.

As for their biological function (Figure 6), more upregulated than downregulated transcripts related to secondary metabolism and photosynthesis were found in the compatible interaction. Regarding to processes related to biotic and abiotic stress, only up-regulated genes were detected. Noteworthy are the genes encoding the Hsp70 chaperone and other proteins related to defensive response, such as PR-1a or a Glucan endo-1,3-beta-glucosidase 3, and a protein related to hypersensitive response (HR-like lesion-inducing protein-related). In contrast, more downregulated than upregulated transcripts were associated with transport-related processes or were grouped into the protein category (including protein synthesis, modification, or degradation) or the RNA category, which includes transcription regulation.

#### 2.3.3. Transcripts Differentially Expressed at 2 dpi Only in the Incompatible Interaction

At the early phase, 341 transcripts were differentially expressed in the incompatible interaction and not in the compatible one (Figure 5). Of them, the expression level of 156 transcripts (up-regulated) was between 2 and 24 times higher in infested than in uninfested Motelle plants. The 185 down-regulated transcripts were expressed between 2 and 14 times less in infested plants than in plants without infestation.

As in the compatible interaction, most transcripts corresponded to genes with unknown function (Figure 6). In addition, other functional categories with a high number of differential genes were obtained. The RNA category (including transcriptional processing, binding, and regulation) was highlighted, represented by several transcription factors, including the WRKY and MYB families, known for their involvement in defence processes, several zinc finger stress associated proteins and a calmodulin-binding protein related to defence response. A number of transcripts related to the metabolism of hormones were also found, among them, proteins involved in defence through the ethylene signal transduction pathway, other related to signalling processes such as Ca^2+^ carried out by calmodulin, or grouped in the protein category (including synthesis, modification and degradation). Genes in the miscellaneous category were also abundant, mostly down-regulated. Up-regulated genes, among which those codifying PRPs (pathogenesis-related proteins), mostly represented the category of biotic stress. Interestingly, Mapman did not include enzymes such as endo-1,3-β-glucosidase in this category, which belongs to the PR-2 class, according to van Loon et al. (2006) [39]. The cell wall category had a higher number of up-regulated transcripts, (including XTHs (Xyloglucan xyloglucosyl transferases and Xyloglucan endotransglucosylase/hydrolases), than down-regulated ones. In contrast, the secondary metabolism category consisted of a larger number of repressed than overexpressed transcripts. Finally, the abiotic stress category was represented only by down-regulated genes, including those encoding small chaperones (sHSP).

### 2.4. Similarities and Differences Between Compatible and Incompatible Interactions in the Late Phase of Whitefly Infestation (12 dpi)

**Figure 7:**
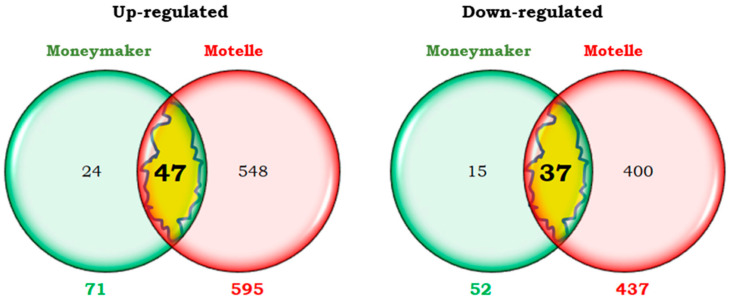
Venn diagram showing the number of transcripts differentially expressed in the tomato-*Bemisia tabaci* compatible (Moneymaker) and incompatible (Motelle) interactions at 12 dpi. The overlap are transcripts equally found in both interactions. Up-regulated represent transcripts more expressed in infested than in non-infested (control) plants. Down-regulated represent transcripts less expressed in infested than in non-infested (control) plants. Only transcripts with statistically significant relative expression values (FDR < 0.05) and fold change (FC) ≥ 2 (up) or ≤−2 (down) are included.

The numbers of both, up- and down-regulated tomato transcripts at 12 days post-infestation (12 dpi) with *B. tabaci* are shown by means of Venn diagrams in Figure 7. A greater number of transcripts were differentially expressed in the incompatible (1032) than in the compatible interaction (123). In both interactions, more up-regulated than down-regulated transcripts were detected (Figure 7). Only 84 of the differential transcripts were common to both compatible and incompatible interactions, as most of them were exclusive of one or the other interaction.

#### 2.4.1. Transcripts Differentially Expressed at 12 dpi in Both Compatible and Incompatible Interactions

A total of 47 transcripts were found to be up-regulated in both interactions (Figure 7), accounting for approximately 8% of those up-regulated in the incompatible interaction and 66% in the compatible. They included genes related to cell wall and defence response, such as glucan endo-1,3-beta-glucosidase.

Data from the 37 common down-regulated transcripts were similar: they represented 8.5% of the total number of transcripts down-regulated in the incompatible interaction and 71% in the compatible. Of these, several genes related to the stress response that encode small chaperones (sHSP) can be highlighted. The intensity of both up- and down- regulation was generally higher in the case of the incompatible interaction (fold change ranging between −47.50 and 42.90) than in the compatible interaction (fold change ranging between −4.48 and 6.07).

#### 2.4.2. Transcripts Differentially Expressed at 12 dpi Only in the Compatible Interaction

Only 39 transcripts were differentially expressed exclusively in the compatible interaction but not in the incompatible one: 24 up-regulated and 15 down-regulated transcripts (Figure 7). The expression level of the 24 overexpressed transcripts ranged from 2 to 5-fold higher in infested than in non-infested Moneymaker plants. The expression level of the 15 exclusively repressed transcripts in the compatible interaction was 2–4 fold lower in infested than in non-infested plants.

**Figure 8:**
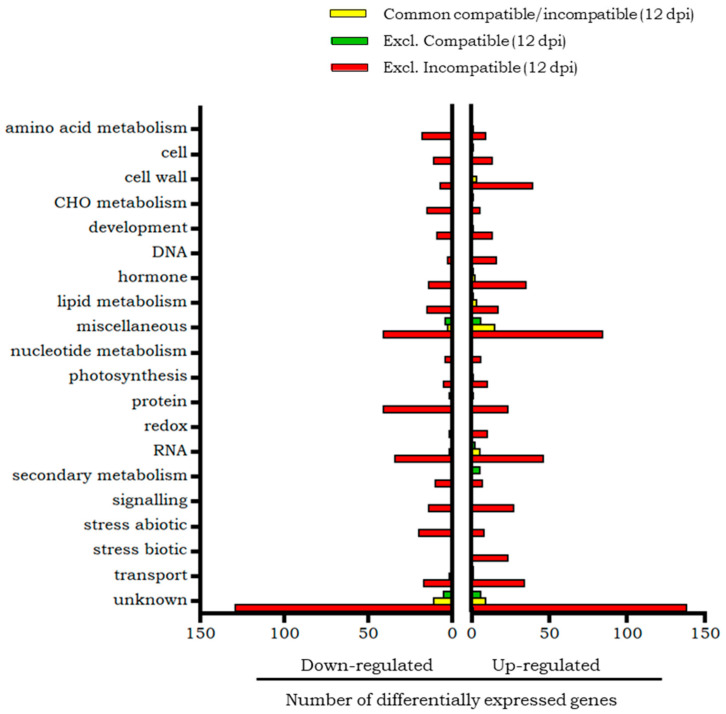
Functional classification by Mapman of the differential transcripts at 12 dpi, exclusive of the compatible interaction (green), exclusive of the incompatible interaction (red) and those in common (yellow), according to the biological processes in which they intervene.

According to the Mapman analysis of biological processes (Figure 8), some of the 24 transcripts that were overexpressed only in the compatible interaction were related to secondary metabolism and, more specifically, to flavonoid metabolism. Among the 15 transcripts exclusively repressed in the compatible interaction, the gene for the enzyme cellulose synthase 3 was found.

#### 2.4.3. Transcripts Differentially Expressed at 12 dpi Only in the Incompatible Interaction

The total number of transcripts differentially expressed in the incompatible interaction at 12 dpi but not in the compatible interaction at the same time point was 948 (Figure 7). Of them, the expression level of the 548 overexpressed transcripts was between 2 and 50 times higher in infested than in uninfested Motelle plants. The 400 transcripts exclusively repressed in the incompatible interaction were expressed between 2 and 86 times less in infested than in uninfested plants.

The RNA category was made up of a similar number of overexpressed transcripts, including the WRKY family and CONSTANS protein, and repressed transcripts, including the transcription factor MADS-box (Figure 8).

The cell wall, hormone metabolism, signalling and DNA categories had a greater number of overexpressed than repressed transcripts. In the cell wall category, the genes that code for expansins, and XTH stand out. The hormone metabolism category included the gene that codes for the DELLA protein GAI (involved in both salicylic acid and jasmonic acid signalling pathways). The signalling category included an herbivore elicitor-regulated protein, a calmodulin, a leucine-rich repeat receptor-like protein related to defence and MAPK3. The DNA category grouped histone-encoding genes, whose expression seems to be induced after infection with pathogens.

The categories including genes involved in the metabolism of amino acids, carbohydrates and secondary metabolism had a greater number of repressed than overexpressed transcripts, highlighting the repression of the ELI3 protein. The protein category also grouped a greater number of repressed than overexpressed transcripts, but among the latter, the genes encoding protease inhibitors should be highlighted. Likewise, in the category of abiotic stress, a greater number of repressed than overexpressed genes was obtained, highlighting the presence of several chaperones, related to the response to stress; while in the category of biotic stress, more overexpressed than repressed genes were found. This category includes numerous PRs, a proteinase inhibitor, and the proteins encoded by *Ve1* and *Ve2* genes, responsible for resistance to *Verticillium dahliae* in tomato. Among those repressed genes related to biotic stress, the enzyme NADPH oxidase should be highlighted. Several overexpressed genes related to stress were not included in this category by Mapman, such as stress responsive A/B barrel domain-containing proteins or a viroid-inducible proteinase inhibitor ii. Similarly, stress related genes not included in this category by Mapman were repressed, such as a gene related to response to abcisic acid and water deprivation.

## 3. Discussion

Trying to improve knowledge on molecular mechanisms involved in the tomato resistance to *B. tabaci*, we previously studied the basal differences in tomato transcriptome between uninfested plants carrying or lacking the *Mi-1* gene (Motelle and Moneymaker, respectively), and how these baseline differences were modified following infestation with *B. tabaci* [10]. However, in the present work, the compatible and incompatible interactions have been separately considered, obtaining in each case (Moneymaker or Motelle, respectively), the tomato transcripts with differential expression after *B. tabaci* infestation with respect to non-infested plants of the same cultivar.

A greater number of differentially expressed transcripts was obtained during the incompatible interaction than in the compatible one (in the two phases studied) as it has been described in the case of nematodes [25]. Furthermore, the expression differences were greater in the incompatible interaction, especially at 12 dpi. Since only a few transcripts were up- or downregulated in both interactions, the overlap in the responses was considered very low, compared to what was published for other plant-pathogen/insect systems [25,35,36,40,41,42,43].

When gene expression was compared between the two time points of each separate interaction, percentages of differential transcripts common to both phases were very low. In the compatible interaction, 3% of the upregulated and 4% of the downregulated transcripts were the same at 2 and 12 dpi, while these figures were 6% and 11% in the incompatible interaction, which indicates that the responses triggered in the tomato plant in these two phases of the infestation are radically different. This seems logical since adult females and/or immature individuals of *B. tabaci* exert a different pressure on the plant in each stage of the interaction, so that it would be expected that the plant response in each phase reflects the differences in the insect’s activity. In both interactions, the number of differentially expressed genes was lower in the early than in the late infestation stage. Conversely, other authors found that N1 activity produced fewer changes in tomato gene expression than female feeding and oviposition in the compatible interaction with *B. tabaci* [29]. Differences in methodology may account for this discrepancy: different tomato cultivars, number of adults, evaluation moments, temperature and *B. tabaci* biotype. The interaction plant-whitefly varies depending on the biotype assessed, resulting in different plant response and infestation levels [4,44,45,46,47]. However, the main difference lies in the data collection and analysis system. We have used the Affymetrix platform with 9200 transcripts, while in the other study [29] 244 genes were used, selected by subtractive suppression hybridization (SSH) and for their involvement in resistance responses to insects and pathogens.

The role of differentially expressed genes, grouped into functional categories, will be discussed below. In the case of the large number of genes that Mapmam did not associate with any category, their role within the corresponding biological process will be discussed, according to our knowledge.

### 3.1. Signalling

Only few transcripts related to signalling processes showed differential expression in the compatible interaction. On the contrary, during the first phase of the incompatible interaction, *B. tabaci* adult feeding and oviposition on *Mi-1-*carrying tomato produced the overexpression of several calmodulins and the repression of two copines. Calmodulins are Ca^2+^ sensors involved in the signalling of this secondary messenger [48,49] that interact with some WRKY transcription factors [50]. Ca^2+^ sensors, and WRKY transcription factors, were transcriptionally upregulated after salicylic acid (SA) treatment in tomato [51]. This agrees with the participation of SA in the *Mi-1* mediated resistance to *B. tabaci* [52], as it has been demonstrated also for aphids and nematodes [15,25,53]. The role of calmodulins is essential in the regulation of plant responses to diverse biotic stress [54], and it has been described in defence processes in numerous interactions [55,56,57], including the *Mi-1* mediated resistance to nematodes [25]. Results of the present work provide additional support for the role of calmodulin in the *Mi-1* mediated resistance to *B. tabaci*. Regarding copines, it has been suggested that this protein family play an important role in plant disease resistance [58], and it represents the pathway of universal transduction for Ca^2+^ signalling, since copines are capable of interact with different target proteins [59]. The repression of copines in the early phase of the incompatible interaction of tomato with *B. tabaci* could indicate a disruption of Ca^2+^ signalling. However, this signalling would take place by the calmodulins, overexpressed also at 12 dpi in the incompatible interaction. Interestingly, a differential expression of copines was not reported in the incompatible interaction of tomato with nematodes, although they were included in the array used [25].

On the other hand, MAPK3 was overexpressed in the two phases of the incompatible interaction with *B. tabaci*, although upregulation was stronger in the early phase. However, no differential expression was detected during the compatible interaction. MAPKs play an important role in plant resistance [60,61,62,63,64]. Specifically, the MAPK3 is actively involved in the resistance mediated by the *Pto* gene to *P. syringae* pv. *tomato* [65] and in the resistance mediated by the *Mi-1* gene to aphids [15]. MAPK3 overexpression in resistant plants after infestation with *B. tabaci* agrees with these results and suggests a role for this MAPK in the *Mi-1*-mediated resistance to whiteflies. In other microarray works carried out with *B. tabaci* on *Arabidopsis* or susceptible tomato, other MAPKs were differentially expressed, but no MAPK3 [29,30], but it was not included in the microarrays used in these works. Several of the MAPKs induced by *B. tabaci* in tomato were related to the response to salicylic acid. Again, all previous information is consistent with the participation of SA in the *Mi-1* mediated resistance [52], as pointed out above.

Also in the signalling category, the phi-1 protein was upregulated only in the early phase of the incompatible interaction. Salt stress also induced expression of phosphate induced proteins [66], which suggests some overlapping between abiotic and biotic stresses.

### 3.2. Respiratory Burst

A NADPH oxidase (respiratory burst oxidase homologue (Rboh)) gene was downregulated exclusively in the incompatible interaction, both at 2 and at 12 d after infestation. Rboh enzymes are the key hubs in mediating the systemic Reactive Oxygen Species (ROS) response in plant tissues [67]. In tomato, the repression of Rboh gene translated into a reduction in the accumulation of H_2_O_2_ [68], impairing resistance against *Phytophthora infestans* [69]. In addition, inhibition of NADPH oxidase-dependent ROS synthesis plays an important role during colonization by *Pseudomonas syringae* pv. *tomato* [70]. On the other hand, ROS accumulation in plants can cause cell damage that could induce a hypersensitive response (HR) [71]. HR occurs in *Mi-1* mediated resistance to nematodes [8], but no differential expression of Rboh genes was detected [25]. In a *Mi-1* resistance-breaking population of nematodes, able to avoid inducing HR, several peroxidases were overexpressed, but not Rboh genes [72]. In the case of aphids, a slight accumulation of ROS was detected in *Mi-1* mediated resistance, without HR [9], but Rboh expression was not studied. In the tomato-*B. tabaci* system, HR also does not occur, and it is possible that down regulation of Rboh gene has a role in this. To confirm this, complementary studies would be necessary, such as those carried out on *Arabidopsis* with whiteflies, which indicate that no increased levels of H_2_O_2_ were detected after prolonged interaction with whiteflies [30] or those just mentioned above on tomato with aphids [9].

Related also to respiratory burst is the upregulation of glutaredoxins in the incompatible interaction, reducing the oxidative stress. In particular, a glutathione -S-transferase was upregulated exclusively in the compatible interaction, especially in the early infestation stage. Increase in glutathione-S-transferases is coincident with accumulation of DELLAs (discussed below), which are associated with low ROS levels [73]. *B. tabaci* induced glutaredoxin upregulation in the two phases of the incompatible interaction, as it has been reported in the incompatible interaction tomato-nematodes [25]. Glutaredoxins probably control the oxidative stress in the *Mi-1-*mediated resistance to whiteflies and nematodes, since they are antioxidant enzymes [74,75]. Glutaredoxins showed a similar relative expression in both phases of tomato infestation by *B. tabaci*, suggesting that a moderate increase would be enough to perform its antioxidant function. Differential glutaredoxin expression was also observed in comparisons between Motelle and Moneymaker uninfested tomato plants, a basal difference maintained after *B. tabaci* infestation [10].

### 3.3. Regulation of Transcription

Transcriptional regulation is one of the processes with differential gene expression between compatible and incompatible interactions. Transcription factors (TF) exclusively overexpressed in the 2d-incompatible interaction included Homeobox (HB), Auxin/Indole-3-acetic acid (AUX-IAA) or GRAS, related to plant development [76,77,78,79,80,81]. This overexpression of Homeobox in the incompatible interaction is in accordance with a Homeobox repression in the compatible interaction, both at 2 dpi. The gene encoding PHOR1, a component of the gibberellin signalling pathway [82], was also exclusively overexpressed in the 2d-incompatible interaction. Other transcription factors related to stress signalling, including Activating Protein 2 (AP2), Heat Shock Factor (HSF), MYB, and WRKY [25,33,83,84,85,86,87] showed mostly upregulation in the incompatible interaction, although some were down regulated, such as some AP2. It is worth mentioning that an AP2 gene was also repressed at 2 dpi in the compatible interaction, which indicates a high functional diversity of these TF. A transcription factor of the family Nuclear transcription factor Y (NF-Y), related to fruit ripening [88] was downregulated at 2 dpi in both interactions.

After 12 days in the incompatible interaction, numerous transcription factors (WRKY, MYB, Homeobox, AP2, bHLH, HSF) were also overexpressed. However, also in the compatible interaction overexpression of Homeobox and AP2 genes was detected, and in the incompatible interaction at 12 dpi the transcription factor MADS-box, related to fruit ripening [77] was repressed. Also, in the incompatible interaction at 12 dpi, a CONSTANS interacting protein was upregulated while another CONSTANS interacting protein was downregulated. The CONSTANS protein is a positive regulator of floral induction, which interacts with the TGA transcription factor that regulates SA-dependent defence genes [89]. This is related with the involvement of the SA pathway in the *Mi-1*-mediated resistance to *B. tabaci* [52], and against aphids and nematodes [15,25].

The WRKY transcription factors are associated with transcriptional reprogramming caused by various types of stress in plants [85,90]. In tomato, WRKY are relevant in diverse pathogenic interactions [91,92]. In the present work, feeding and oviposition by adult females of *B. tabaci* did not induce WRKY expression in susceptible tomato plants, a result similar to that previously described in the compatible interaction with the A biotype of *B. tabaci* [29]. However, the expression of WRKY was indeed induced by the activity of the N2-N3 nymphs of *B. tabaci* in susceptible *Arabidopsis* and tomato [29,30], as it was observed in the present study for WRKY6, overexpressed at 12d in both the compatible and the incompatible interactions. On the other hand, WRKY6 was induced only in the incompatible tomato-nematode interaction, while other WRKYs were overexpressed during both the compatible and incompatible interactions [25]. WRKY6 was also upregulated in compatible interaction tomato-aphids, but overexpression of WRKY46 was stronger [21]. In tobacco plants, NtWRKY4, NtWRKY6, and NtWRKY10 were significantly upregulated in whitefly-infested plants or plants treated with salicylic acid [93]. Again, this points out the interaction between WRKYs and SA signalling. According to Coppola et al. [21], WRKY genes are involved in the antagonist interaction between SA and Jasmonic Acid (JA). One of the WRKYs with a role in *Mi-1*-mediated resistance to aphids and nematodes, WRKY72 [94], was not represented on the Affymetrix chip used. Therefore, we cannot know its role in the case of *B. tabaci*, and this deserves further investigation.

### 3.4. Defence Genes

#### 3.4.1. PR-Proteins

It is known that *B. tabaci* feeding on tomato induces Pathogenesis Related (PR) proteins: β-1,3-glucanase, chitinase, peroxidase, P2, and P4 [95]. In the present work, PRs showed a differential expression level depending on the type of interaction and the *B. tabaci* infestation stage. Moreover, PRs are related to hormone signalling, which is discussed below.

The expression of a gene encoding a PR-1 protein which is specifically related to biotic stress processes, was increased exclusively in the early phase of the compatible interaction but never in the incompatible one. PR-1 had been shown to be upregulated in response to *B. argentifolii* second- and third-instar feeding on *Arabidopsis* [30]. On the other hand, the PR-2 protein (glucan endo-1,3-α-glucosidase (GluB) was overexpressed in the compatible and incompatible interactions at 2 and 12 days. However, at 12 days, overexpression was greater in the incompatible interaction (16 fold) than in the compatible interaction (2 fold). Thus, it seems that this protein is related to *Mi-1* resistance in the late infestation phase. The expression of this PR-2 was previously demonstrated to be induced in the incompatible interaction tomato-*P. syringae* pv. *tomato* [96], during the compatible and incompatible tomato-aphid interactions [9] and during the compatible interactions of tomato with *B. argentifolii* [95] and *B. tabaci* [29]. A higher expression in resistant than in susceptible plants has also been observed in the wheat-aphid interaction [97].

#### 3.4.2. Heat Shock Proteins

HSPs (chaperones and proteases) play a crucial role in protecting plants against stress [98,99]. To focus on the tomato plant, it was demonstrated that the chaperone HSP90 has a role in the *Mi-1.2*-mediated resistance to aphids and nematodes [17], as well as to the whitefly *B. tabaci* [18]. In the present study, the differential expression of chaperones was highly variable among them. So, chaperone Hsp70 was upregulated in tomato at 2 dpi of the compatible interaction with *B. tabaci*, while other chaperones were down regulated at 2 dpi in the incompatible interaction. With regard to the later phase, some chaperones were down regulated at 12 dpi in both the compatible and incompatible interactions, although the down regulation was stronger in the incompatible one. It seems that, although there are processes common to biotic and abiotic stresses [100], the activity of whiteflies represses rather than overexpresses those genes related to abiotic stress. In addition, downregulation of HSPs is stronger in the incompatible interaction, indicating that this repression is important for *Mi-1* resistance. This decrease in the expression of abiotic stress proteins was previously observed in response to viruliferous and non-viruliferous whiteflies on *B. tabaci*-susceptible tomato plants [101]. This decrease could promote the chances of plant colonization by *B. tabaci* [32].

#### 3.4.3. Secondary Metabolites

From the obtained results, one of the groups with the highest number of overexpressed genes in the compatible interaction is related to secondary metabolism, mainly of flavonoids, whose production follows activation of the phenylpropanoid pathway [102]. Flavonoids play an important role in plant defence and are activated in response to biotic and abiotic stress [103]. On the other hand, the enzyme isoflavone reductase, involved in the flavonoid pathway, was overexpressed in the incompatible interaction of tomato with *B. tabaci*, both at 2 and 12 dpi. This enzyme was upregulated also after attack by the brown grasshopper *Nilaparvata lugens* on resistant rice plants [104]. The coincidence of these results could be related to the phloem feeding mode shared by both insects. Conversely, the incompatible interaction of cassava with the bacteria *X. axonopodis* pv. *manihotis* (Xam) resulted in the repression of the isoflavone reductase [105].

The protein ELI3 was repressed exclusively in the incompatible interaction of the present study, both at 2 and 12 days after whitefly infestation. ELI3 is a cinnamyl alcohol dehydrogenase (CAD) protein [106], involved in lignin biosynthesis, whose expression is lower in Motelle than in Moneymaker tomato at 2 days after *B. tabaci* infestation [10]. CAD enzymes are associated with defence in several plant-pathogen systems. In tomato, CAD1 was involved in resistance to *Ralstonia solanacearum* [107] and ELI3 has an important role in the resistance response to *Cladosporium fulvum* [108]. However, in the tomato-nematode interaction, ELI3 levels are overexpressed in both the compatible and incompatible interactions [25]. This supports the existence of different carrying *Mi-1* tomato responses against nematodes and *B. tabaci*.

### 3.5. Genes Putatively Involved in Biotic Stress

#### 3.5.1. Cell Wall Genes

*B. tabaci* infestation affected expression of genes related to cell wall, which is not surprising, as pathogen and insect attack commonly alter composition of cell wall [32,109]. However, alterations observed in the present work were different in the two interactions and moments of infestation. Some genes were repressed exclusively in the compatible interaction, at 2 dpi (endo-β-1,4-D-glucanase) or at 12 dpi (cellulose synthase). Endo-1,4-β-D-glucanases are required for normal wall assembly and cell growth [110,111]. It is possible that *B. tabaci* feeding on the susceptible cultivar Moneymaker releases proteins that degrade plant enzymes to avoid a structural modification of the cell wall that hinders its feeding. Regarding the down-regulation of cellulose synthase in the susceptible tomato cultivar, this enzyme was also repressed in the compatible interaction whiteflies-*Arabidopsis* [30], which suggests a basal defence mechanism in tomato and *Arabidopsis*. Increased pathogen resistance was observed in an *Arabidopsis* mutant with constitutive inhibition of cellulose synthase [112]. Also in *Arabidopsis*, the inhibition of cellulose synthase subunits related to the formation of the secondary cell wall, can activate specific defence pathways against pathogens [113].

In the present study, the overexpression of genes related to the cell wall occurred mainly in the incompatible interaction and especially at a late infestation stage. Earlier (2 dpi), *B. tabaci* adult feeding resulted in upregulation of the gene encoding XTH, but also of genes encoding pectinesterases. This has been reported also in other interactions, mainly incompatible, such as aphid resistant apple and sorghum [32]. At a later infestation stage, tomato protection against *B. tabaci* should increase in the resistant plant, since additional genes encoding expansins, extensins, pectinesterases, polygalacturonase inhibitory proteins (PGIP) and pectate lyases were also upregulated. Pectate lyases are primarily released by pathogens to degrade the cell wall of plants [114] but also regulate plant development and ripening [115] and participate in the defence system of *Arabidopsis* against different types of stress [116]. PGIPs are located in the plant cell wall maintaining a defensive strategy against attack by pathogens and insects, as they act against polygalacturonases (PG) released by them, which degrade cell wall [117,118,119]. The induction of PGIPs has been reported mainly in the incompatible plant-pathogen interaction [117]. However, these inhibitory proteins were not induced during the incompatible tomato-nematode interaction [25]. All these structural modifications of the cell wall during the late phase of incompatible interaction, also observed in other incompatible interactions with insects [32,120], suggest that cell wall regulation plays an important role in the *Mi-1* tomato resistance to *B. tabaci*.

#### 3.5.2. Hormone

The tomato gene encoding the phospholipase β2 (PLDβ2) enzyme was repressed at the early infestation stage of both compatible and incompatible interactions with *B. tabaci*, which does not allow us to associate it with either type of interaction in particular. Mapman classified this gene in the category of lipid metabolites, but PLDβ2 is a member of the phospholipase D family, which is involved in different plant processes, including stress and hormone signalling [121,122]. In other pathosystems, it has been seen that this enzyme is related to plant resistance: So, PLDβ2 is upregulated in the tomato-spider mite system during the first 5 days after infestation [123]. Also, another phospholipase D (PLDdelta) was upregulated in resistant tomato at a very early stage (15 min) of infestation with *Ralstonia solanacearum,* although it was immediately downregulated [107]. Moreover, PLDβ is necessary in *Arabidopsis* for pattern-triggered immunity (PTI) [124]. Unfortunately, PLDβ2 was not included in the study of the tomato-nematode interactions [25]. Thus, we cannot yet know if both systems (tomato-*B. tabaci* and tomato-nematodes) share the role of this enzyme, although further work is being carried out by our group to try to answer this and other questions.

As in other processes, transcriptional regulation of hormone signalling was most intensely modified in the incompatible interaction, and especially at a later infestation stage. During *B. tabaci* adult feeding at 2 dpi, ethylene response genes were overexpressed, while those involved in the response to auxins and abscisic acid were generally repressed, with some exceptions. For example, two Small Auxin-up RNA (SAUR) genes were overexpressed. Mapman did not assign SAUR genes to stress categories, but these genes in tomato are involved in the response to aluminum stress [125]. The induction of ethylene response has also been observed in tomato during the feeding of aphids [126] and nematodes [25]. Further on, N1 feeding induced the expression of a large number of genes involved in the metabolism of different hormones. For example, genes involved in the synthesis of ethylene stand out, among which are the genes that encode the enzymes ACC (1-aminocyclopropane-1-carboxylic acid synthase) and ACO (1-aminocyclopropane-1-carboxylic acid oxidase) [127,128]. Ethylene accumulates in various plants after pathogen or insect attack, although it does not always have the same role in compatible or incompatible interactions (reviewed by van Loon et al. [39]). Ethylene-dependent responses are amplified by signalling cascades through transcription factors, including ERF1 (Ethylene Response Factor 1) [129], also overexpressed during the late phase of the incompatible interaction. In tomato, simulated herbivory increased the abundance of other ERF, such as ERF16 [130].

In addition, it is known that the activation of ethylene signalling reduces the levels of gibberellins, promoting the accumulation of DELLA proteins that slow down plant development [73,131]. This is consistent with the overexpression of DELLA-type proteins found in the work reported here, specifically the GAI protein, which belongs to the GRAS TF gene family, comprehensively analyzed in tomato [78]. All this indicates that, after prolonged exposure to *B. tabaci*, plants carrying the *Mi-1* gene overexpress the GAI protein, so that plant resources are allocated to defence rather than to plant development. Although the basal expression of DELLA proteins is lower in uninfested Motelle than in Moneymaker plants, it is noteworthy that this difference in expression is reduced after infestation with *B. tabaci* [10], which supports that DELLA proteins are important in the resistance mediated by the *Mi-1* gene.

GRAS proteins are involved in the regulation of defensive responses in tomato, as these proteins are induced in resistant tomato plants after pathogen attack [23,132]. Also, GRAS tomato genes are upregulated upon mycorrhization [133]. On the other hand, DELLA proteins are also related to defence against abiotic stress, such as salt stress in *Arabidopsis* [73]. In tomato, DELLA protein PROCERA acts reducing water loss by increasing abscisic acid (ABA) sensitivity [134]. Overexpression of DELLA proteins has also a role in avoiding oxidative stress because it parallels an increase in the levels of peroxidases and glutathione S-transferases, which regulate the accumulation of ROS. Achard et al. [73] reported this in response to biotic and abiotic stress in *Arabidopsis.* In tomato, SlGRAS10 enhances osmotic potential, flavonoid biosynthesis, and ROS scavenging [135].

DELLAs have a role in regulating SA signalling to sustain a balance between defence and plant growth [136]. We have found that SA is involved in the *Mi-1* mediated resistance to *B. tabaci* [52], and the present data confirm the importance of the SA-signalling pathway in such a resistance. At an early infestation stage in the incompatible interaction, several PR proteins and MAPKs related to SA response were induced, and upregulation of genes involved in the synthesis of SA occurred in the late infestation stage, together with an overexpression of PRs, phenylalanine ammonia lyase (PAL) and MAPK dependent on SA. It is relevant to point out, however, that PR1, one of the most important marker genes in SA-mediated disease resistance to pathogens [137] was only upregulated in the compatible interaction with *B. tabaci*. Other works have demonstrated the involvement of SA in the *Mi-1* mediated resistance to aphids and nematodes [25,53]. In addition, we have shown that *B. tabaci* fitness was similar in the *Mi-1* plants with altered SA route as in plants lacking the *Mi-1* gene [52]. Interestingly, during the late phase of the incompatible interaction, genes involved in JA synthesis were also overexpressed, such as AOS (allene oxide synthase) and FAD3 (fatty acid desaturase 3). This agrees with the results obtained during the incompatible tomato-aphid interaction, where the expression of PR proteins regulated by SA was induced [9]. It was concluded that aphids would activate both signalling pathways. However, other studies showed that the participation of SA is necessary for *Mi-1*-mediated resistance to aphids [15] but not that of JA [138]. In addition, SA is not exclusive of incompatible interactions, as *B. tabaci* induced in tomato responses involving SA, and suppressed plant defence responses involving JA [139]. SA is also involved in a tomato-aphid compatible interaction, with a possible antagonistic crosstalk between the SA- and JA-signalling pathways [21]. Different works with whiteflies in *Arabidopsis* [30] and tomato [29,95,139,140] have shown antagonistic communication between both routes.

Finally, it is possible that Gly-Asp-Ser-Leu (GDSL)-lipases are involved in the *Mi-1*-mediated resistance to *B. tabaci* through their relationship with hormone signalling. Overexpression of one GDSL-lipase was much more intense in the incompatible interaction than in the compatible at 12 dpi. Plant GDSL Esterases/Lipases have a role in the response to biotic stress in diverse plant pathogen interactions, and they are related to SA, JA, and ethylene hormones [141]. In the incompatible interaction wheat-Hessian fly, increased abundance of GDSL-motif lipase/hydrolase mRNA was reported [142].

#### 3.5.3. Other Stress Genes

Our results show differential expression of other genes related to plant stress. Firstly, some universal stress proteins were strongly down-regulated in the incompatible interaction at 12 dpi, although not much additional information about them was available. Moreover, the gene encoding the transmembrane protein Bax-Inhibitor-1 (BI-1) was upregulated in compatible and incompatible interactions at 2 dpi. BI-1 is an evolutionary conserved protein that represents an ancient cell death regulator that potentially regulates programmed cell death in all eukaryotes [143]. Infection with the pathogen *P. syringae* in *Arabidopsis* caused the overexpression of this protein [144], and BI-1 enhanced disease resistance, showing a higher SA and higher PR1 expression level [137]. In other studies, transformed barley plants overexpressing BI-1 had a reduced susceptibility to *Fusarium graminearum* [145], and it suppressed cell death in rice caused by *Magnaporthe grisea* [146]. On the other hand, BI-1 is required for full susceptibility of barley to powdery mildew [147]. In *Arabidopsis*, BI-1 interacts with calmodulin [148], and also with aquaporin plasma membrane intrinsic protein TaPIP-1 in response to SA signals [137]. In the present system tomato-whitefly, calmodulins were only overexpressed in the incompatible interaction at 2 dpi. During the later phase of infestation, the BI-1 protein was not differentially expressed during the incompatible interaction. This could indicate that the BI-1 protein rapidly increases its expression after *B. tabaci* infestation, but a prolonged increase is not required in the *Mi-1*-mediated resistance to whiteflies. Regarding a possible interaction of BI-1 with aquaporins, the aquaporin PIP-1 was not overexpressed in either the compatible or incompatible interaction with *B. tabaci*. Several tonoplast intrinsic proteins (TIPs) and PIPs were overexpressed exclusively at 12 dpi of the incompatible interaction, while another TIP was strongly repressed.

The Plant Cadmium Resistance 2 (*PCR2*) gene was also upregulated in both interactions at 2 dpi. However, at 12 dpi it was only overexpressed in the incompatible interaction and more intensely than at 2 dpi. Thus, it seems related to *Mi-1* mediated resistance, although its expression did not differ in compatible and incompatible interactions tomato-nematodes [25]. This gene is upregulated in tomato after ethylene treatment in the abscission area [149], and it is upregulated in *Trichoderma* primed tomato [150].

The enzyme XEG inhibitor protein (XEGIP) was down regulated in both compatible and incompatible interactions, but only 12 days after infestation. This enzyme inhibits pathogenic beta-1,4-endoglucanases (XEG) that degrade cell walls in a pathogen infected plant, and upregulation of XEGIP was observed after pathogen attack, wound or abiotic stress [149,151]. However, according to the present results, it is possible that XEGIP is not able to inhibit *B. tabaci* endoglucanases because it is specific for XEG and not for other enzymes, as previously pointed out [151].

The enzyme acid phosphatase-1 (Aps-1) was upregulated in the late phase of the incompatible interaction. This enzyme is involved in defence processes, mainly to insects, and it is closely linked to the *Mi-1* gene on chromosome 6 [152]. During the comparison of the transcriptomes of cultivars Motelle and Moneymaker after infestation with *B. tabaci*, this enzyme was also more expressed in the carrying-*Mi-1* Motelle plants [10].

*Ve1* and *Ve2* genes were upregulated exclusively in the incompatible interaction, *Ve2* at 2 dpi and then both in the late stage. These genes are in the Ve locus of the tomato chromosome 9 and *Ve1* provides resistance against some strains of the genus *Verticillium* [153,154]. The signalling cascade of the *Ve1* gene partially overlaps with the signalling cascade mediated by Cf resistance genes in tomato [153]. These proteins contain a leucine-rich repeat region (LRR) [154], as the *Mi-1* gene [12]. Thus, the present results with *B. tabaci* suggests that *Ve* and *Mi-1*-mediated resistance could be related, with a possible overlapping between both signalling pathways.

At the early infestation stage, the short-chain dehydrogenase TIC32 was down regulated in both compatible and incompatible interactions. Tic32 is a component of the Tic complex that participates in the process of translocation of the inner envelope of chloroplasts, making it essential for their biogenesis [155,156]. Down regulation of Tic32 has been reported also in heat-stressed pea [157], indicating a similarity in plant responses to biotic and abiotic stress. Similarly, we found upregulation of a HXXXD-type acyl-transferase family protein in the incompatible interaction at 12 dpi. These proteins are involved in cold stress in grapevine [158] and tea [159].

It was remarkable the intense upregulation of beta-hydroxysteroid dehydrogenase at 12 dpi in the incompatible interaction. Although to our knowledge no involvement with plant resistance has been described for this gene, it has been suggested that the 3-beta-hydroxysteroid dehydrogenase family may play a key role in the intumescence development in tomato [160].

A stress responsive A/B barrel domain-containing protein was upregulated in the incompatible interaction at 12 dpi. Stress-responsive dimeric A/B barrel domains characterize the DABB proteins, involved in plant defence against biotic and abiotic stress [161,162,163]. The *A. thaliana* gene coding for a stress response A/B barrel domain-containing protein has antifungal properties [164].

Finally, several genes encoding proteins with Domains of Unknown Function (DUFs) were differentially expressed in both compatible and incompatible interactions, but much more intensely in the incompatible interaction. There are DUF genes that are activated in *Arabidopsis* upon pathogen challenge, and likely playing important roles in disease resistance, and has been referred to as the Pathogen and Abiotic stress response, cadmium tolerance, and Disordered Region-containing (PADRE) domain [165]. Our work suggests DUF genes are likely relevant also for insect resistance.

## 4. Materials and Methods

### 4.1. Plant Material

The tomato cultivars Moneymaker and Motelle were used to characterize gene expression in the compatible and incompatible interactions with *B. tabaci*, respectively. These cultivars are quasi-isogenic, differing only by the presence of a 650 kb introgressed region from *Solanum peruvianum* containing the *Mi-1* gene in Motelle cultivar [166,167].

Surface sterilized seeds were germinated in autoclaved vermiculite (number 3, Projar, Valencia, Spain) and kept under controlled conditions at 25 °C, 16:8 (L:O) and 70% R.H. Plants were watered when needed, while every 15 days they were nourished with a complex 20-20-20 (Nutrichem 60; Miller Chemical, Hanover, PA, USA) at a concentration of 3 g·L^−1^. Plants were grown for 8 weeks (8–9 true leaves) prior to *B. tabaci* infestation.

### 4.2. Whitefly Infestations

Plants were infested with adult females of the MED (Mediterranean) species of *B. tabaci*, reared for several generations on tomato cultivar Marmande at 24 °C, 16:8 h (L:O) and 60% H.R. Infestation was performed following the methodology described in [10]. Briefly, each of three well-developed leaflets from the upper part of the tomato plant was placed into a cage made up from a 50 mL Falcon tube. A lateral hole was drilled in those cages to introduce 30 female whiteflies per leaflet. Empty cages made of Falcon tubes were placed in other plants (control plants).

### 4.3. Sampling

Samples of tomato leaflets were taken at two moments after infestation, which were chosen taking into account the duration of the development phases of *B. tabaci* at 25 °C [168]. According to this, two days after infestation the development stage of *B. tabaci* was adult/egg and at 12 days the immature stages (nymphs) were present.

- 2 dpi (days post infestation): two days after the introduction of the females in the cages with the leaflets. At this time, the females have laid eggs and thus the effect of feeding and oviposition is assessed.
- 12 dpi: twelve days after infestation. At this time, most of the individuals are N1 nymphs, which inject the stylet at different points into the leaves until the suitable spot is found to settle and continue development as sessile nymphs.

Twelve plants of each tomato variety were sampled at each time point (2 and 12 dpi): 6 plants were infested with *B. tabaci*, while 6 remained uninfested (control plants). After two days, empty cages and cages with whiteflies were carefully removed, and infested leaflets were checked to ensure that all *B. tabaci* adults were removed. Then, three biological samples of each cultivar were collected from infested and uninfested plants. Each biological replicate was made up to six leaflets, each one collected from a different plant of the same cultivar and treatment (infested or uninfested). These samples corresponded to 2 dpi and the sampled plants were discarded. A similar process was followed in other plants at 12 dpi. The sampled leaflets were quickly frozen with liquid nitrogen and kept at −80 °C until RNA extraction and microarray analysis.

### 4.4. Sample Processing for Microarray Hybridization

Samples were ground using a mortar and RNA extraction was performed using Trizol Reagent (Thermo Fisher Scientific, Waltham, MA, USA) and purified with RNeasy mini kit “clean-up” protocol (Qiagen, Hilden, Germany). The integrity of the extracted RNA was determined by means of the Bioanalyzer 2100 (Agilent Technologies, Santa Clara, CA, USA). cDNA synthesis, production of cRNA biotin-labelled and its hybridization to Affymetrix^®^ arrays was performed as described in our previous work [10].

### 4.5. Data Analysis

The data obtained from the microarrays were analyzed through BABELOMICS (http://babelomics.bioinfo.cipf.es/) accessed on 1 July 2011 [169] applying the Robust Multi-array Average (RMA) [170] to adjust the background, normalize, and log-transform values. Raw *p* values were adjusted for multiple hypotheses testing using the false discovery rate (FDR) method [171]. Genes with a fold-change in expression ≥ 2 or ≤−2 and FDR < 0.05 were considered as differentially expressed.

The VENNY programme version 2.1 [172] was used to compare the lists of previously selected genes and to identify the genes shared in the different gene lists.

Descriptions of the genes and target sequences corresponding to GeneChip probesets were obtained from Affymetrix, Tomato Annotations Release 36 (NetAffx Analysis Center). Target sequences were also used in BLAST (version 2.2.27) searches of their corresponding tomato genes (version SL3.0 and Annotation ITAG4.1) in Sol Genomics database [173].

Functional analysis of differentially expressed genes was performed using the Mapman software version 3.1.0. Analysis of GO term enrichment was performed with the Singular Enrichment Analysis (SEA) tool in AgriGO V2.0 [174]. Significant terms (*p*-value < 0.05, Fisher’s exact test) were selected for plotting with the ‘ggplot2’ R library [175].

## 5. Conclusions

In this work we have found a very important differential gene expression in tomato plants during both the compatible (Moneymaker) and incompatible (Motelle) interactions with *B. tabaci*, detectable as early as two days after infestation by whitefly adults. Transcriptional reprogramming intensifies over time when immature stages of the insect are feeding on the plant at 12 days after infestation, both in the resistant and susceptible tomato varieties. However, and more importantly, the presence of the *Mi-1* resistance gene in cv. Motelle causes a more intense cascade of transcriptional reprogramming after the attack of *B. tabaci*, compared to Moneymaker plants without this gene. This process of overexpression or repression of genes ultimately will trigger biological processes that end up hindering the development of the whitefly.

Although many of the tomato transcripts differentially expressed after *B. tabaci* infestation were assigned to genes with unknown function or included in the miscellaneous group, other transcripts were related to relevant plant defence genes. Thus, some genes were involved in signalling, respiratory burst, regulation of transcription and hormonal processes. Genes encoding PRs and HSPs, secondary metabolites and cell wall genes, amongst others, also showed differential expression.

This information provides very valuable knowledge to better understand the mechanisms involved in the *Mi-1*-mediated resistance of tomato against *B. tabaci* and paves the way for new research to help complete the scheme of the susceptibility and defence responses to this very important insect pest.

## Data Availability

Data is contained within the article or Supplementary Material.

## Supplementary Materials

The following supporting information can be downloaded at: https://www.mdpi.com/article/10.3390/plants14071054/s1, Table S1: Lists of genes differentially expressed during the tomato/*B. tabaci* interactions. Figure S1: Gene Ontology term enrichment of differential transcripts.
