# Supplementary material for: Over Time Changes in the Transcriptomic Profiles of Tomato Plants with or Without Mi-1 Gene During Their Incompatible or Compatible Interactions with the Whitefly Bemisia tabaci
Source: Plants (Basel). 2025 Mar 28;14(7):1054. doi: 10.3390/plants14071054 (PMC11990454; doi:10.3390/plants14071054)
Supplement: Supplementary file 1 [file plants-14-01054-s001.zip › Supplementary Material Figure S1.pdf]

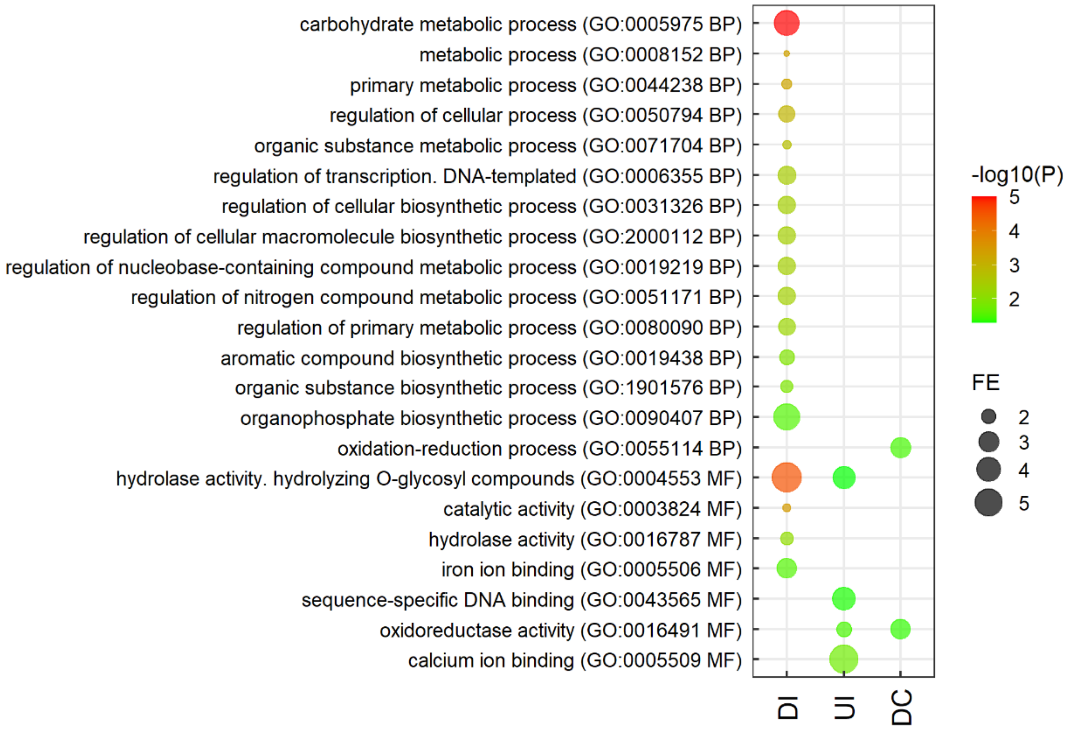

Figure S1. Gene Ontology (GO) term enrichment of the differential transcripts exclusive of the incompatible (DI, down-regulated incompatible; UI up-regulated incompatible) and compatible (DC, down-regulated compatible) interactions. Dot size represents fold-enrichment (FE) of the GO-term in the corresponding gene list over the complete genome and the colour scale the associated P-value in –log10 scale. BP, Biological Process; MF, Molecular Function.
